# A SYSTEMATIC REVIEW OF RANDOMIZED CONTROLLED TRIALS OF COGNITIVE TRAINING INTERVENTIONS IN OLDER ADULTS

**DOI:** 10.1093/geroni/igz038.2434

**Published:** 2019-11-08

**Authors:** Ayse Malatyali, Carol Reive, Christine Williams

**Affiliations:** Christine E Lynn College of Nursing, Florida Atlantic University, Boca Raton, Florida, United States

## Abstract

There has been a growing interest in cognitive training interventions for their potential effect of maintaining and promoting cognitive functioning in older adults. Rapid and significant changes in technology has had a significant impact on the design and assessment methods of cognitive training interventions. Investigating changes in brain networks and blood markers are relatively new approaches and sparsely examined in the literature. The purpose of this systematic literature review is to analyze the effect of cognitive training interventions on brain networks, blood markers and associated cognitive performance of healthy older adults. We conducted a comprehensive literature search on four databases, following PRISMA guidelines. Initially, 2426 citations were retrieved, and 251 full-text publications were evaluated in detail for eligibility. Fourteen randomized control trials were included in this review. Functional imaging analysis of brain networks showed significant activity changes primarily in the Default Mode Network. These changes were associated with improvement in memory, learning, attention, and affective performances. Also, there were activity changes in the Central Executive Network that were associated with improvement in reasoning, attentional control, innovative thinking, and processing speed. Training-induced changes have been observed in the brain-derived neurotrophic factor levels and the markers of antioxidative and anti-inflammatory regulatory mechanisms. Improvement in attention and memory performances were significantly related to these changes. Limitations of the studies included methodological inconsistencies, sampling issues, and the lack of long-term follow up assessment. Cognitive training appears to promote improvement and maintenance of cognitive functioning in healthy older adults.

